# Food Insufficiency Following Discontinuation of Monthly Child Tax Credit Payments Among Lower-Income US Households

**DOI:** 10.1001/jamahealthforum.2022.4039

**Published:** 2022-11-11

**Authors:** Zoe Bouchelle, Aditi Vasan, Molly Candon, Chén C. Kenyon

**Affiliations:** 1 National Clinician Scholars Program, Perelman School of Medicine, University of Pennsylvania, Philadelphia; 2 Department of Pediatrics, Perelman School of Medicine, University of Pennsylvania, Philadelphia; 3 PolicyLab, Children’s Hospital of Philadelphia, Philadelphia, Pennsylvania; 4 Leonard Davis Institute of Health Economics, Perelman School of Medicine, University of Pennsylvania, Philadelphia; 5 Department of Psychiatry, Perelman School of Medicine, University of Pennsylvania, Philadelphia; 6 Department of Health Care Management, Wharton School, University of Pennsylvania, Philadelphia

## Abstract

**Importance:**

The 2021 expanded Child Tax Credit provided advance monthly payments to many US families with children from July through December 2021 and was associated with a reduction in food insufficiency. Less is known about the effect of the discontinuation of monthly payments.

**Objective:**

To assess whether the discontinuation of monthly Child Tax Credit payments was associated with subsequent changes in food insufficiency among lower-income US households with children.

**Design, Setting, and Participants:**

This population-based cross-sectional study used data from the Household Pulse Survey, a recurring online survey of US households conducted by the US Census Bureau, from January 2021 to March 2022. This study estimated difference-in-differences regression models for households making less than $50 000, less than $35 000, and less than $25 000 annually, adjusting for demographic characteristics and state of residence. The estimation sample of households making less than $50 000/y included 114 705 responses, representing a weighted population size of 27 342 296 households.

**Exposures:**

Receipt of monthly Child Tax Credit payments, as measured by living in a household with children during the period of monthly payments from July through December 2021.

**Main Outcomes and Measures:**

Household food insufficiency, as measured by a respondent indicating that there was sometimes or often not enough food to eat in the household in the previous 7 days.

**Results:**

Among 114 705 households making less than $50 000/y, respondents were predominantly female (57%); White (71%); not of Hispanic, Latino, or Spanish origin (79%); had high school or equivalent education (38%); and were unmarried (70%). Following the discontinuation of monthly Child Tax Credit payments, food insufficiency in US households with children increased by 3.5 percentage points (95% CI, 1.4-5.7 percentage points) among households making less than $50 000/y, 4.9 percentage points (95% CI, 2.6-7.3 percentage points) among households making less than $35 000/y, and 6.2 percentage points (95% CI, 3.3-9.3 percentage points) among households making less than $25 000/y. These estimates represent a relative increase in food insufficiency of approximately 16.7% among households making less than $50 000/y, 20.8% among households making less than $35 000/y, and 23.2% among households making less than $25 000/y.

**Conclusions and Relevance:**

In this population-based cross-sectional study, discontinuation of monthly Child Tax Credit payments in December 2021 was associated with a statistically significant increase in household food insufficiency among lower-income households, with the greatest increase occurring in the lowest-income households.

## Key Points

**Question:**

Was the discontinuation of advance monthly Child Tax Credit payments in December 2021 associated with changes in food insufficiency among lower-income US households?

**Findings:**

In this population-based cross-sectional study using data from a recurring online survey of US households conducted by the US Census Bureau, discontinuation of advance monthly Child Tax Credit payments was associated with an increase in food insufficiency of approximately 6.2 percentage points among households with children making less than $25 000/y.

**Meaning:**

Advance monthly Child Tax Credit payments may have served as a buffer against food insufficiency among lower-income US households with children.

## Teaser

This cross-sectional study assesses whether the discontinuation of monthly Child Tax Credit payments in December 2021 was associated with subsequent changes in food insufficiency among lower-income US households with children.

## Introduction

Many families with children living in the US experienced substantial economic hardship as a result of the COVID-19 pandemic and the associated economic recession. In response, the 2021 American Rescue Plan^1^ temporarily restructured the Child Tax Credit (CTC) to provide additional financial support to families with children. The expanded CTC (eCTC) increased the monetary value of the credit, expanded access to the full credit for low- and no-income households, and permitted a portion of the credit to be distributed in advance monthly payments from July to December 2021.^2^

As policy makers debate the future size, eligibility, refundability, and structure of the CTC, it is critical to understand the health and economic effects of these payments. While there may be numerous downstream benefits of this program on children’s health,^3^ one key measure of the program’s immediate effect on children’s health and well-being is the association between these payments and families’ access to food. Approximately 29.5% of all households with children in the US experienced food insecurity in April and May 2020, the first months of the COVID-19 pandemic.^4^ Monthly eCTC payments may have been a beneficial adjunct to government nutrition benefit programs and community-based food resources in helping these families afford adequate and nutritious food during the pandemic and associated economic recession.

When these payments were discontinued, children in these households may have been at increased risk of food insecurity and associated adverse health outcomes, including developmental delay, behavioral problems, and school absenteeism, as well as decreased access to preventive care.^5,6,7,8^ Lower-income households are more likely to experience inadequate access to food and are also at highest risk of being excluded from the full CTC benefit as the program reverts back to its previous structure in 2022.^9^

Just as the implementation of the monthly eCTC program provided an opportunity to study the effects of a per-child, monthly cash benefit policy in the US, discontinuation of the program provides an opportunity to study the effect of *not* having such a policy, particularly for lower-income households with children. Thus, the objective in this cross-sectional study was to use a quasi-experimental design to estimate the association of discontinuation of monthly eCTC payments on food insufficiency among US households with children, with a particular focus on lower-income families. To do this, we compared food insufficiency among households with and without children in the 3 lowest income brackets available in our data source, performing separate analyses for households making less than $50 000,less than $35 000, and less than $25 000 annually, which correspond to households making less than the US median household income (approximately $67 521 in 2020).^10^

## Methods

### Study Sample and Assignment of Exposure

We used data from the Household Pulse Survey, a recurring, cross-sectional online survey of US households conducted by the US Census Bureau. This study was deemed nonhuman participant research by the Children’s Hospital of Philadelphia Institutional Review Board and did not require ongoing oversight. The study meets the Strengthening the Reporting of Observational Studies in Epidemiology (STROBE) reporting guideline for cross-sectional studies.

We used data from 22 survey waves, with data collection beginning January 6, 2021, and ending March 14, 2022. Each wave of data collection of the Household Pulse Survey lasts approximately 2 weeks. Earlier waves collected and released data in 2-week increments, while later waves spaced data collection to a 2-week-on and 2-week-off schedule. The specifics of data handling, imputation, and survey weighting are published by the US Census Bureau (a summary of imputed values is provided in eTable 1 in the Supplement).^11,12^ Response rates for the survey from January 2021 to March 2022 ranged from 5.4% to 7.9%.^13^

In this analysis, we included participants aged 18 to 65 years to focus on families with working-age adults. Survey data with a minimum partial completeness are included in the public-use files, and variables included later in the survey have differential missingness (a summary of missing data is provided in eTables 2 and 3 in the Supplement).^14^ Among all households with respondents aged 18 to 65 years, those who identified as Black or African American or Hispanic, Latino, or Spanish origin were more likely to have missing data for income and food insufficiency. Because we performed a complete case analysis when adjusting for covariates, this resulted in the exclusion of households with missing data for the primary independent and dependent variables, excluding disproportionately more households with a primary respondent that identified as Black or African American or Hispanic, Latino, or Spanish origin.

### Exposure

Consistent with prior literature on this subject,^15^ households with children were defined as the exposed group, with the exposure to monthly eCTC payments occurring during the survey waves beginning July 21, 2021, and ending December 13, 2021. Households without children were defined as the unexposed group because they were not eligible for the CTC.

### Outcome

The primary outcome was a binary indicator of household food insufficiency. This was based on the survey item “Getting enough food can also be a problem for some people. In the last 7 days, which of these statements best describes the food eaten in your household?” Households were considered food insufficient if they responded either “Sometimes not enough food to eat” or “Often not enough food to eat” and food sufficient if they responded either “Enough of the kinds of food [I/we] wanted to eat” or “Enough, but not always the kinds of food [I/we] wanted to eat.”^16^ This question is drawn from the more extensive 18-item scale developed by the US Department of Agriculture,^17^ with both the item and scale validated at the household level.^15,16^

We adjusted for covariates including gender at birth (female or male), age group (18-24, 25-44, or 45-65 years), race (Asian, Black or African American, White, or any other race alone or in combination), ethnicity (Hispanic, Latino, or Spanish origin or non–Hispanic, Latino, or Spanish origin), educational level (less than high school, high school or equivalent, some college or 2-year degree, or 4-year degree or graduate-level degree), marital status (married or unmarried), number of adults in the household (1, 2, or ≥3), number of children in the household (0, 1, 2, or ≥3), Supplemental Nutrition Assistance Program receipt (yes or no), receipt of free food in previous 7 days (yes or no), annual income bracket (<$25 000, $25 000-$34 999, or $35 000-$49 999), and state of residence. The race and ethnicity data are based on respondents’ self-report, and categories represent the data in the files made publicly available by the US Census Bureau. The Household Pulse Survey does not collect additional information from respondents who identify as “any other race alone or in combination.” While race and ethnicity are social constructs, we included these variables in our analysis given prior research showing that Black or African American and Hispanic children are more likely to be food insecure than White children, likely related to disproportionate poverty prevalence and effects of systemic racism and discrimination on these children and their families.^18^

### Statistical Analysis

**Figure 1.**
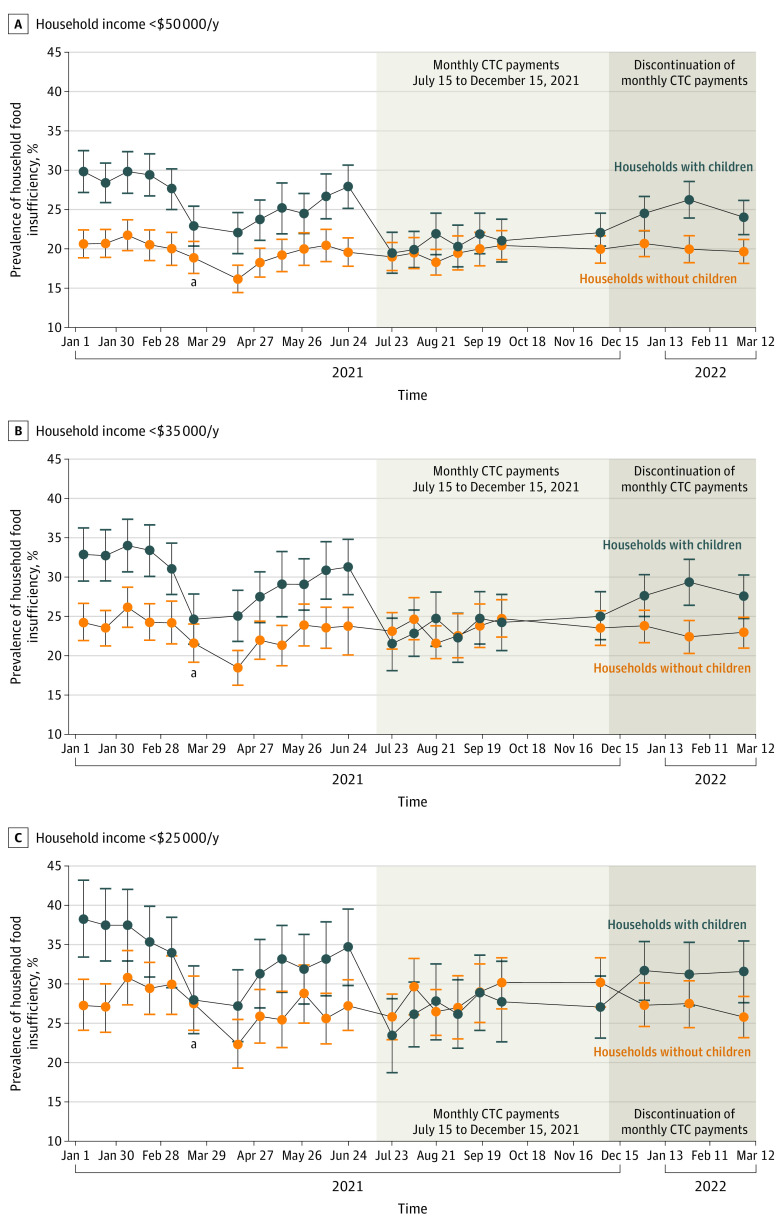
US Household Food Insufficiency From January 2021 to March 2021, Stratified by Annual Income Unadjusted, weighted prevalence of US household food insufficiency per analysis of Household Pulse Survey data from the US Census Bureau. Responses were weighted using household survey weights divided by the 22 waves in the sample. Respondents were 18 to 65 years of age. Respondents with missing data for primary dependent and independent variables in difference-in-differences analysis were excluded. The x-axes plot equally spaced dates with data points plotted on first date of data collection of each 2-week survey wave. Error bars represent 95% CIs. Unshaded regions denote survey waves preceding implementation of monthly Child Tax Credit (CTC) payments, which are not included in difference-in-differences analysis. Light gray regions denote survey waves during the period of monthly CTC payments, which are included in difference-in-differences analysis. Dark gray regions denote survey waves after the period of monthly CTC payments, which are also included in difference-in-differences analysis. ^a^Period during which a large share of lump-sum tax credits and the March 2021 Economic Impact Payments were distributed.

To examine the longitudinal trends in household food insufficiency in each income group, we plotted the unadjusted, weighted prevalence of household food insufficiency for exposed and unexposed households across 22 survey waves between January 6, 2021, and March 14, 2022 (Figure 1). Next, we restricted the survey to the 10 waves that occurred during and after the monthly eCTC payments, with data collection beginning July 21, 2021, and ending March 14, 2022, to isolate the removal of the monthly eCTC payments. For each income group, we compared the baseline characteristics of exposed and unexposed households (Table 1 and eTables 4 and 5 in the Supplement).

**Table 1.** Baseline Characteristics of US Households With Annual Income <$50 000, July 2021-March 2022, Stratified by Exposure

Characteristics	No. (weighted %)^a^		
	Total sample (n = 114 705)^b^	Households without children (n = 75 414)^b^	Households with children (n = 39 291)^b^
Age category, y			
18-24	6700 (9)	5348 (11)	1352 (6)
25-44	46 709 (46)	23 641 (36)	23 068 (63)
45-65	61 296 (44)	46 425 (53)	14 871 (30)
Gender at birth			
Female	78 140 (57)	47 933 (51)	30 207 (66)
Male	36 565 (43)	27 481 (49)	9084 (34)
Race			
Asian	4636 (4)	2997 (4)	1639 (4)
Black or African American	14 918 (18)	8040 (15)	6878 (24)
White	86 767 (71)	59 526 (75)	27 241 (64)
Any other race alone or in combination^c^	8384 (7)	4851 (6)	3533 (8)
Ethnicity			
Hispanic, Latino, or Spanish origin	16 797 (21)	8681 (16)	8116 (28)
Not of Hispanic, Latino, or Spanish origin	97 908 (79)	66 733 (84)	31 175 (72)
Educational attainment			
Less than high school	5464 (12)	2591 (9)	2873 (17)
High school or equivalent	23 240 (38)	14 183 (37)	9057 (40)
Some college or 2-y degree	49 919 (33)	31 929 (34)	17 990 (33)
4-y degree or graduate level	36 082 (16)	26 711 (20)	9371 (11)
Marital status			
Unmarried	80 739 (70)	57 246 (76)	23 493 (60)
Married	33 966 (30)	18 168 (24)	15 798 (40)
2020 household income			
<$25 000	44 040 (42)	29 686 (43)	14 354 (42)
$25 000-$34 999	31 710 (28)	20 444 (27)	11 266 (28)
$35 000-$49 999	38 955 (30)	25 284 (30)	13 671 (30)
Total adults in household			
1	41 240 (33)	30 924 (38)	10 316 (25)
2	47 572 (42)	29 536 (39)	18 036 (46)
≥3	25 893 (25)	14 954 (22)	10 939 (29)
Total children in household			
0	75 414 (62)	75 414 (100)	0
1	18 520 (17)	0	18 520 (44)
2	11 984 (12)	0	11 984 (31)
≥3	8787 (10)	0	8787 (25)
SNAP receipt			
Yes	27 979 (29)	13 406 (20)	14 573 (43)
No	86 726 (71)	62 008 (80)	24 718 (57)
Free food receipt in previous 7 d			
Yes	11 460 (11)	5902 (9)	5558 (16)
No	103 245 (89)	69 512 (91)	33 733 (84)
Survey wave dates			
Jul 21-Aug 2, 2021	10 587 (10)	7037 (10)	3550 (10)
Aug 4-Aug 16, 2021	10 876 (9)	7195 (10)	3681 (9)
Aug 18-Aug 30, 2021	10 938 (9)	7209 (9)	3729 (9)
Sept 1-Sept 13, 2021	10 118 (10)	6737 (10)	3381 (9)
Sept 15-Sept 27, 2021	9533 (10)	6373 (10)	3160 (9)
Sept 29-Oct 11, 2021	9057 (9)	6116 (9)	2941 (9)
Dec 1-Dec 13, 2021	11 285 (11)	7575 (11)	3710 (11)
Dec 29, 2021-Jan 10, 2022	14 083 (11)	8918 (11)	5165 (12)
Jan 26-Feb 7, 2022	13 776 (11)	8852 (11)	4924 (11)
Mar 2-Mar 14, 2022	14 452 (10)	9402 (10)	5050 (11)

Abbreviation: SNAP, Supplemental Nutrition Assistance Program.

^a^ Responses were weighted using household survey weights divided by the 10 waves in the sample.

^b^ Unweighted observation frequency.

^c^ The Household Pulse Survey does not collect additional information from respondents who identify as “any other race alone or in combination.”

Again using the 10 waves that occurred during and after the monthly eCTC payments, we then estimated difference-in-differences models to measure the association of discontinuation of monthly eCTC payments with the outcome of interest, food insufficiency, for each income group. Difference-in-differences is a quasi-experimental technique that isolates the relationship between policy changes and outcomes across exposed (ie, treatment) and unexposed (ie, comparison) groups by accounting for secular trends.^19,20^ We estimated multivariable linear probability models with food insufficiency as the primary outcome.^21,22^ The model included group fixed effects, time fixed effects, and a binary treatment variable (the interaction term) that varied at the group and time levels (eMethods 1 in the Supplement).^23^ We estimated unadjusted and adjusted models (with adjusted models including the covariates described previously) for 3 income strata: less than $50 000, less than $35 000, and less than $25 000 annually.

A key identifying assumption in the difference-in-differences models is that outcomes in exposed households would have continued along their same trajectories as the unexposed households in the absence of the exposure. To assess this parallel-trends assumption, we visually inspected trends in the unadjusted, weighted prevalence of the primary outcome within exposed and unexposed households during the pretreatment period, beginning with the July 21, 2021, survey wave, for each income group (Figure 1). We also performed statistical testing to examine whether the linear trends in the primary outcome were parallel between exposed and unexposed groups during the pretreatment period, beginning with the July 21, 2021, survey wave, for each income group (eMethods 2 in the Supplement). Because the final survey waves did not include information about whether recipients had ever previously received monthly eCTC payments, we were unable to use self-reported receipt of eCTC payments as the primary exposure in the difference-in-differences analysis.

We performed sensitivity analyses to assess the robustness of the results. We estimated the association between the discontinuation of monthly eCTC payments and household food insufficiency using a difference-in-differences model that allowed for the associations between exposure and outcome to vary over time (also known as an event study specification).^23^ This approach is less prone to bias when the association between exposure and outcome changes over time (eMethods 3 in the Supplement).

Two-sided *t* tests or χ^2^ tests were used to test for statistically significant differences in the exposed and unexposed groups. All *P* values were from 2-sided tests and results were deemed statistically significant at *P* < .05. For all models, we used household survey weights divided by the number of waves in the sample. Standard errors were clustered at the state level to allow for intragroup correlation within a state. All analyses were conducted using Stata/MP software, version 17.0 (StataCorp). Data analyses were performed between January and May 2022.

## Results

Weighted individual demographics and household socioeconomic characteristics of households making less than $50 000/y are summarized in Table 1. The estimation sample had 114 705 responses, representing a weighted population size of 27 342 296 households. The sample was predominantly female (57%); White (71%); not of Hispanic, Latino, or Spanish origin (79%); had high school or equivalent education (38%); and was unmarried (70%). Weighted individual demographics and household socioeconomic characteristics of households making less than $35 000/y and less than $25 000/y are summarized in eTables 4 and 5 in the Supplement, respectively.

Figure 1 plots the unadjusted, weighted prevalence of the primary outcome (household food insufficiency) within exposed and unexposed households by survey wave for each income group. Figure 1 shows that for each income group, the unadjusted, weighted prevalence of food insufficiency among households with children was higher than that of households without children from January to July 2021, at which time the prevalence of food insufficiency among the 2 groups converged. Beginning in December 2021, the prevalence of food insufficiency among the 2 groups diverged again such that households with children experienced a higher prevalence of food insufficiency. Visual inspection of the shaded portions of the graph (denoting the survey waves during and after the period of monthly eCTC payments, which are included in difference-in-differences models) suggests that the parallel-trends assumption is satisfied. Formal statistical testing also demonstrated parallel trends (eMethods 2 and eTable 6 in the Supplement).

Results of the unadjusted and adjusted difference-in-differences models are summarized in Table 2. Following the discontinuation of the advance monthly eCTC payments, food insufficiency in US households with children increased by 3.5 percentage points (95% CI, 1.4-5.7 percentage points) among households making less than $50 000/y, 4.9 percentage points (95% CI, 2.6-7.3 percentage points) among households making less than $35 000/y, and 6.2 percentage points (95% CI, 3.3-9.3 percentage points) among households making less than $25 000/y. Relative to the mean household food insufficiency during the period of the monthly payments, these estimates represent a relative increase in food insufficiency of approximately 16.7% among households making less than $50 000/y, 20.8% among households making less than $35 000/y, and 23.2% among households making less than $25 000/y.

**Table 2.** Difference-in-Differences Estimates of the Association Between Food Insufficiency and Discontinuation of Monthly Child Tax Credit Payments, Stratified by Annual Income

Annual income	Model	Difference-in-differences estimate, percentage point change (95% CI)	*P* value
<$50 000	Unadjusted^a^	3.3 (1.1-5.6)	.004
	Adjusted^b^	3.5 (1.4-5.7)	.002
<$35 000	Unadjusted^a^	5.0 (2.5-7.4)	<.001
	Adjusted^b^	4.9 (2.6-7.3)	<.001
<$25 000	Unadjusted^a^	6.2 (3.1-9.3)	<.001
	Adjusted^b^	6.2 (3.3-9.3)	<.001

^a^ Unadjusted model does not include covariate adjustment. Respondents with missing data for primary dependent and independent variables in adjusted difference-in-differences analysis were excluded. Standard errors are clustered at the state level.

^b^ Adjusted model includes covariate adjustment for birth, age group, race, ethnicity, educational level, marital status, number of adults in the household, number of children in the household, Supplemental Nutrition Assistance Program receipt, receipt of free food in the previous 7 days, annual income, and state of residence. Standard errors are clustered at the state level.

**Figure 2.**
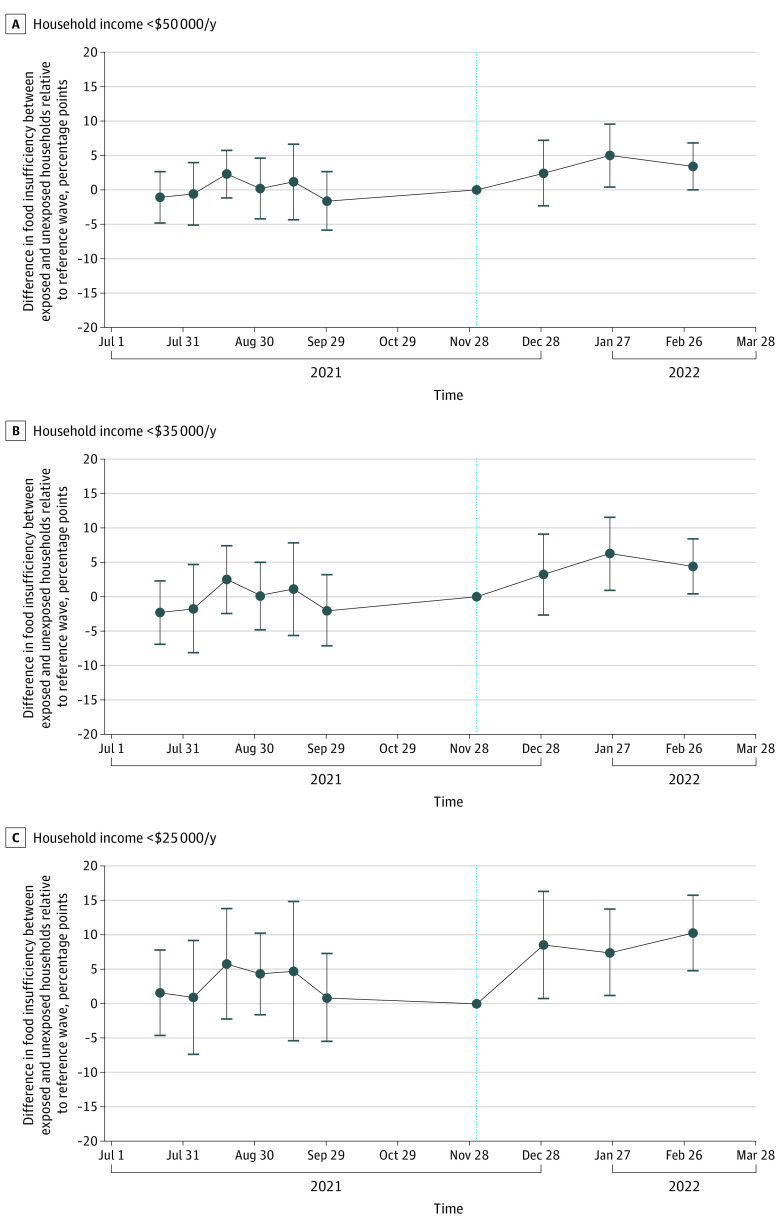
Difference-in-Differences Estimates of the Association Between Discontinuation of Monthly Child Tax Credit Payments and Household Food Insufficiency, Stratified by Annual Income Adjusted difference-in-differences estimates (the absolute adjusted difference between exposed [households with children] and unexposed [households without children] groups) for the primary outcome, household food insufficiency. Models allow for the associations between exposure and outcome to vary over time by using an event study specification. Responses were weighted using household survey weights divided by the 10 waves in the sample. Respondents were 18 to 65 years of age. Respondents with missing data for primary dependent and independent variables in difference-in-differences analysis were excluded. The x-axes plot equally spaced dates with data points plotted on first date of data collection of each 2-week survey wave. Error bars represent 95% CIs. The vertical dashed lines represents the reference survey wave (December 1-13, 2021), which occurred immediately prior to the policy change (discontinuation of monthly Child Tax Credit payments on December 15, 2021).

Figure 2 displays the difference-in-differences estimates over time for each income group, with each point on the y-axis reflecting the difference in household food insufficiency between exposed and unexposed households relative to the survey wave prior to discontinuation of advance monthly eCTC payments, denoted by the vertical dashed line (eTable 7 in the Supplement).

## Discussion

Following the discontinuation of monthly eCTC payments, the percentage of US households with children experiencing food insufficiency increased, with larger increases in food insufficiency among households with lower income levels. These estimates represent a relative increase in food insufficiency of approximately 16.7% among households making less than $50 000/y, 20.8% among households making less than $35 000/y, and 23.2% among households making less than $25 000/y. Based on food insufficiency estimates in households with children with incomes less than $50 000/y from the Household Pulse Survey in March 2022 (6 376 168 households), we estimate that the discontinuation of the monthly eCTC payments translated to 215 619 more households with children experiencing food insufficiency. These results provide evidence that the monthly eCTC payments may have served as a key buffer against household food insufficiency for lower-income children and families.

These findings are consistent with prior research focused on the implementation of the eCTC. Research using Household Pulse Survey data found that the first monthly eCTC payments were associated with a 3.7–percentage point decline in food insufficiency among all households with children^15^ and a 7.5–percentage point decline in food insufficiency among households with children earning less than $35 000/y.^24^ A study using the Department of Agriculture’s 18-item US Household Food Security Survey Module over the first 3 months of the eCTC also found a 7.1% decrease in the prevalence of families experiencing very low food security.^25^ These earlier results are further supported by evidence that the majority of families used their monthly payments for basic household expenses like food, clothing, shelter, and utilities.^25,26,27,28^ The present findings expand on this prior work by using quasi-experimental methods to study the consequences of the discontinuation of the monthly eCTC payments, which has not been previously reported. The increase in food insufficiency after the discontinuation of the monthly eCTC payments suggests that low-income families may have benefited from ongoing monthly eCTC payments even as the US entered a state of pandemic recovery.

These results are particularly relevant to the policy debate around the structure of the CTC. Prior to the 2021 expansion, approximately 25 million children in low-income families did not receive the full per-child CTC because their parents lacked earnings or had earnings that were too low.^9,29^ The 2021 reforms made the eCTC fully refundable and removed the minimum income requirement, meaning that they did not exclude no- and low-income households from being eligible for the full credit benefit (regardless of the dollar amount of taxes owed). The households in this study experiencing the greatest increase in food insufficiency following discontinuation of monthly payments—those making less than $25 000/y—are also the households most likely to be excluded from the full CTC benefit as the benefit reverts to its previous structure, which is tied to household earnings, in 2022.

Finally, the present findings are relevant to the broader policy discussions around the benefits of per-child cash benefits paid to families to help offset the costs of raising children, commonly referred to as child allowance programs. The US is an outlier among other high-income nations in that it has never had a permanent federal child allowance program.^30,31^ In fact, in 2016 the US Congress commissioned the National Academies of Sciences, Engineering, and Medicine to provide a nonpartisan, evidence-based report on the most effective means for reducing child poverty by half in the next 10 years.^32^ The 2019 report, *A Roadmap to Reducing Child Poverty*, found that among many potential policy solutions, a $250 per child per month ($3000 per year) child allowance policy—similar in structure to the 2021 eCTC reforms—was likely to make the biggest effect on child poverty.^32^ These types of monthly payments are also supported by evidence in the economics literature that suggests that compared with lump-sum disbursements, periodic payments may help to reduce household income volatility.^33,34,35^

### Limitations

Interpretation of these findings is subject to several limitations. First, the US Census Bureau Household Pulse Survey was designed to quickly and efficiently deploy data collected on how people’s lives were affected by the COVID-19 pandemic. Overall response rates were low, and the survey is subject to nonresponse bias. For example, missing data for income and food insufficiency were more likely among respondents identifying as Black or African American or of Hispanic, Latino, or Spanish origin. We expect that exclusion of observations with missing data in these fields may underrepresent these demographic groups. Second, not all households with children received the eCTC and, as such, this amounts to an “intention-to-treat analysis.” We expect that this may bias results toward the null. Third, because this analysis extends into March 2022, it is possible that some households (both with and without children) had received their lump-sum tax credits. However, we expect that if this additional support were received it would bias results toward the null because it would likely serve to reduce food insufficiency. Fourth, households with children and those without may differ on several measured and unmeasured factors not included in the data set or accounted for in the study design (ie, the differential effects between households with and without children due to loss of in-person childcare or school closures). Fifth, there were many other pandemic-era policy changes to federal nutrition programs to support US households. These include implementation of the novel Pandemic Electronic Benefit Transfer and grab-and-go school meal programs,^36^ as well as policy waivers and associated adaptations to the Supplemental Nutrition Assistance Program and the Special Supplemental Nutrition Program for Women, Infants, and Children.^37^ However, to our knowledge, there were no substantial changes to these programs that occurred concurrently with discontinuation of the eCTC.^38^ Finally, because the monthly payments expired in mid-December, the discontinuation of payments occurred when many children were transitioning to holiday vacations and thus not in school. It is possible that this timing may have affected households with and without children differently, as low-income families with children may rely on free or reduced-price school meals for children, which are less available during instructional breaks.^39^ Extending this analysis into March helps control for this potential effect.

## Conclusions

In this population-based, cross-sectional study, discontinuation of monthly expanded CTC payments in December 2021 was associated with a statistically significant increase in household food insufficiency among lower-income households, with the greatest increase occurring in the lowest-income households. Nonrestricted cash transfer programs like the monthly eCTC represent a promising approach to mitigating income instability and reducing food insufficiency among families with children. With the expiration of the monthly eCTC in 2021, additional policies aimed at mitigating the health and economic effects of poverty on children and families are urgently needed.

## Notes

Supplement. **eTable 1.** Summary of Values Imputed by the U.S. Census Bureau **eTable 2.** Summary of Missing Data, Stratified by Race **eTable 3.** Summary of Missing Data, Stratified by Ethnicity **eTable 4.** Baseline Characteristics of U.S. Households with Annual Income <$35,000, July 2021-March 2022, Stratified by Exposure **eTable 5.** Baseline Characteristics of U.S. Households with Annual Income <$25,000, July 2021-March 2022, Stratified by Exposure **eMethods 1.** Description of Difference-in-Differences Analysis **eMethods 2.** Description of Parallel Trends Testing **eTable 6.** Parallel Trends Testing for Difference-in-Differences Analyses, Stratified by Annual Income **eMethods 3.** Description of Event Study Specification **eTable 7.** Adjusted Difference-in-Differences Estimates of Association Between Food Insufficiency and Discontinuation of Monthly Child Tax Credit Payments, Event Study Specification
eReferences
