# Errors in Figure 1

**DOI:** 10.1001/jamahealthforum.2022.4770

**Published:** 2022-12-09

In the Original Investigation titled “Food Insufficiency Following Discontinuation of Monthly Child Tax Credit Payments Among Lower-Income US Households,”^1^ published November 11, 2022, shading in all panels of Figure 1 has been corrected to indicate that the final monthly Child Tax Credit payments occurred on December 15, 2021. This article has been corrected.
