# Supplementary material for: Food Insufficiency Following Discontinuation of Monthly Child Tax Credit Payments Among Lower-Income US Households
Source: JAMA Health Forum. 2022 Nov 11;3(11):e224039. doi: 10.1001/jamahealthforum.2022.4039 (PMC9652756; doi:10.1001/jamahealthforum.2022.4039)
Supplement: Supplement. — eTable 1. Summary of Values Imputed by the U.S. Census Bureau eTable 2. Summary of Missing Data, Stratified by Race eTable 3. Summary of Missing Data, Stratified by Ethnicity eTable 4. Baseline Characteristics of U.S. Households with Annual Income <$35,000, July 2021-March 2022, Stratified by Exposure eTable 5. Baseline Characteristics of U.S. Households with Annual Income <$25,000, July 2021-March 2022, Stratified by Exposure eMethods 1. Description of Difference-in-Differences Analysis eMethods 2. Description of Parallel Trends Testing eTable 6. Parallel Trends Testing for Difference-in-Differences Analyses, Stratified by Annual Income eMethods 3. Description of Event Study Specification eTable 7. Adjusted Difference-in-Differences Estimates of Association Between Food Insufficiency and Discontinuation of Monthly Child Tax Credit Payments, Event Study Specification eReferences [file jamahealthforum-e224039-s001.pdf]

Supplemental Online Content

Bouchelle Z, Vasan A, Candon M, Kenyon CC. Food insufficiency following discontinuation of monthly child tax credit payments among lower-income US households. *JAMA Health Forum*. 2022;3(11):e224039. doi:10.1001/jamahealthforum.2022.4039

eTable 1. Summary of Values Imputed by the U.S. Census Bureau
eTable 2. Summary of Missing Data, Stratified by Race
eTable 3. Summary of Missing Data, Stratified by Ethnicity
eTable 4. Baseline Characteristics of U.S. Households with Annual Income <$35,000, July 2021-March 2022, Stratified by Exposure
eTable 5. Baseline Characteristics of U.S. Households with Annual Income <$25,000, July 2021-March 2022, Stratified by Exposure
eMethods 1. Description of Difference-in-Differences Analysis
eMethods 2. Description of Parallel Trends Testing
eTable 6. Parallel Trends Testing for Difference-in-Differences Analyses, Stratified by Annual Income
eMethods 3. Description of Event Study Specification
eTable 7. Adjusted Difference-in-Differences Estimates of Association Between Food Insufficiency and Discontinuation of Monthly Child Tax Credit Payments, Event Study Specification
eReferences

This supplemental material has been provided by the authors to give readers additional information about their work.

© 2022 Bouchelle Z et al. *JAMA Health Forum.*

**eTable 1.** Summary of Values Imputed by the U.S. Census Bureau		
**Characteristic**	**Imputed**	**Not Imputed**
Birth year, No. (Weighted %)	13875 (1.0)	1549189 (99.0)
Gender at birth, No. (Weighted %)	15026 (1.1)	1548038 (99.0)
Ethnicity, No. (Weighted %)	37749 (3.1)	1525315 (96.9)
Race, No. (Weighted %)	43363 (4.4)	1519701 (95.6)
Educational attainment, No. (Weighted %)	15831 (1.1)	1547233 (98.9)
Number of persons in the household, No. (Weighted %)	47826 (3.3)	1515238 (96.7)
Number of children in the household, No. (Weighted %)	37500 (2.9)	1525564 (97.1)

Responses weighted using household survey weights divided by the 22 waves in sample.

© 2022 Bouchelle Z et al. *JAMA Health Forum.*

**eTable 2**. Summary of Missing Data, Stratified by Race				
**Characteristic**	**Asian**	**Black or African American**	**White**	**Any Other Race Alone or in Combination**[a]
Annual income, No. (%)	23092 (27.8)	39115 (31.3)	275260 (21.5)	18801 (25.8)
Food insufficiency, No. (%)	11343 (13.7)	19680 (15.8)	122150 (9.5)	9116 (12.5)
Marital status, No. (%)	1031 (1.2)	1704 (1.4)	13540 (1.1)	724 (1.0)
SNAP receipt, No. (%)	12197 (14.7)	21386 (17.1)	135055 (10.5)	9958 (13.7)
Free food receipt in previous 7 days, No. (%)	11597 (14.0)	20634 (16.5)	125806 (9.8)	9480 (13.0)

[a]The Household Pulse Survey does not collect additional information from respondents who identify as "any other race alone or in combination."

© 2022 Bouchelle Z et al. *JAMA Health Forum.*

**eTable 3.** Summary of Missing Data, Stratified by Ethnicity		
**Characteristic**	**Not of Hispanic, Latino, or Spanish origin**	**Hispanic, Latino, or Spanish origin**
Annual income, No. (%)	311323 (22.0)	44945 (30.2)
Food insufficiency, No. (%)	139209 (9.8)	23080 (15.5)
Marital status, No. (%)	14974 (1.1)	2025 (1.4)
SNAP receipt, No. (%)	153773 (10.9)	24823 (16.7)
Free food receipt in previous 7 days, No. (%)	143624 (10.2)	23893 (16.0)

© 2022 Bouchelle Z et al. *JAMA Health Forum.*

**eTable 4.** Baseline Characteristics of U.S. Households with Annual Income <$35,000, July 2021-March 2022, Stratified by Exposure			
	**Total sample**	**Households without children**	**Households with children**
	**N[a]=75750**	**N[a]=50130**	**N[a]=25620**
Characteristics	No. (Weighted %)[b]	No. (Weighted %)[b]	No. (Weighted %)[b]
Age category (years)			
Age 18-24	5163 (10%)	4174 (12%)	989 (7%)
Age 25-44	29817 (45%)	14868 (34%)	14949 (63%)
Age 45-65	40770 (45%)	31088 (53%)	9682 (30%)
Gender at birth			
Female	52313 (59%)	31913 (52%)	20400 (70%)
Male	23437 (41%)	18217 (48%)	5220 (30%)
Race			
Asian	3039 (4%)	2002 (4%)	1037 (4%)
Black or African American	10623 (20%)	5620 (16%)	5003 (26%)
White	56154 (69%)	39008 (74%)	17146 (61%)
Any other race alone or in combination[c]	5934 (7%)	3500 (6%)	2434 (8%)
Ethnicity			
Hispanic, Latino, or Spanish origin	11428 (21%)	5929 (16%)	5499 (29%)
Not of Hispanic, Latino, or Spanish origin	64322 (79%)	44201 (84%)	20121 (71%)
Educational attainment			
Less than high school	4513 (14%)	2178 (11%)	2335 (20%)
High school or equivalent	17053 (40%)	10362 (39%)	6691 (42%)
Some college or 2-year degree	33300 (32%)	21710 (34%)	11590 (30%)
4-year degree or graduate level	20884 (14%)	15880 (17%)	5004 (8%)
Marital status			
Unmarried	56871 (74%)	39936 (80%)	16935 (66%)
Married	18879 (26%)	10194 (20%)	8685 (34%)
2020 household income			
<$25 000	44040 (60%)	29686 (61%)	14354 (59%)
$25 000-$34 999	31710 (40%)	20444 (39%)	11266 (41%)
Household adults (total)			
1	29314 (36%)	21820 (41%)	7494 (28%)
2	29436 (39%)	18529 (37%)	10907 (43%)
>=3	17000 (25%)	9781 (22%)	7219 (29%)

© 2022 Bouchelle Z et al. *JAMA Health Forum.*

Household children (total)			
0	50130 (62%)	50130 (100%)	0 (0%)
1	12216 (17%)	0 (0%)	12216 (44%)
2	7617 (12%)	0 (0%)	7617 (30%)
>=3	5787 (10%)	0 (0%)	5787 (25%)
SNAP receipt (yes or no)			
Yes	23994 (35%)	11991 (26%)	12003 (51%)
No	51756 (65%)	38139 (74%)	13617 (49%)
Free food receipt in previous 7 days (yes or no)			
Yes	9082 (13%)	4897 (11%)	4185 (18%)
No	66668 (87%)	45233 (89%)	21435 (82%)
Survey Wave Dates			
Jul 21-Aug 2, 2021	6981 (10%)	4672 (10%)	2309 (10%)
Aug 4-Aug 16, 2021	7137 (9%)	4706 (9%)	2431 (9%)
Aug 18-Aug 30, 2021	7143 (9%)	4756 (9%)	2387 (9%)
Sept 1-Sept 13, 2021	6669 (10%)	4503 (10%)	2166 (9%)
Sept 15-Sept 27, 2021	6256 (10%)	4196 (10%)	2060 (9%)
Sept 29-Oct 11, 2021	6023 (9%)	4113 (9%)	1910 (9%)
Dec 1-Dec 13, 2021	7576 (11%)	5117 (11%)	2459 (11%)
Dec 29, 2021-Jan 10, 2022	9345 (11%)	5944 (11%)	3401 (11%)
Jan 26-Feb 7, 2022	9036 (10%)	5838 (10%)	3198 (11%)
Mar 2-Mar 14, 2022	9584 (10%)	6285 (10%)	3299 (10%)

[a]Unweighted observation frequency.

[b]Responses weighted using household survey weights divided by the 10 waves in sample.

[c]The Household Pulse Survey does not collect additional information from respondents who identify as "any other race alone or in combination."

Abbreviation: SNAP: Supplemental Nutrition Assistance Program

© 2022 Bouchelle Z et al. *JAMA Health Forum.*

**eTable 5.** Baseline Characteristics of U.S. Households with Annual Income <$25,000, July 2021-March 2022, Stratified by Exposure			
	**Total sample**	**Households without children**	**Households with children**
	**N[a]= 44,040**	**N[a]= 29,686**	**N[a]= 14,354**
Characteristics	No. (Weighted %)[b]	No. (Weighted %)[b]	No. (Weighted %)[b]
Age category (years)			
Age 18-24	3385 (11%)	2768 (13%)	617 (7%)
Age 25-44	16370 (44%)	8129 (32%)	8241 (63%)
Age 45-65	24285 (45%)	18789 (55%)	5496 (29%)
Gender at birth			
Female	30629 (60%)	18909 (52%)	11720 (73%)
Male	13411 (40%)	10777 (48%)	2634 (27%)
Race			
Asian	1676 (4%)	1146 (4%)	530 (3%)
Black or African American	6463 (21%)	3467 (17%)	2996 (28%)
White	32165 (68%)	22824 (73%)	9341 (60%)
Any other race alone or in combination[c]	3736 (8%)	2249 (7%)	1487 (9%)
Ethnicity			
Hispanic, Latino, or Spanish origin	6639 (21%)	3509 (16%)	3130 (29%)
Not of Hispanic, Latino, or Spanish origin	37401 (79%)	26177 (84%)	11224 (71%)
Educational attainment			
Less than high school	3234 (16%)	1606 (12%)	1628 (23%)
High school or equivalent	10439 (41%)	6425 (40%)	4014 (43%)
Some college or 2-year degree	19068 (31%)	12854 (33%)	6214 (28%)
4-year degree or graduate level	11299 (12%)	8801 (16%)	2498 (7%)
Marital status			
Unmarried	35266 (79%)	24890 (84%)	10376 (72%)
Married	8774 (21%)	4796 (16%)	3978 (28%)
Household adults (total)			
1	19010 (40%)	14242 (45%)	4768 (32%)
2	15566 (36%)	9938 (34%)	5628 (39%)
>=3	9464 (24%)	5506 (21%)	3958 (28%)
Household children (total)			
0	29686 (62%)	29686 (100%)	0 (0%)
1	6938 (17%)	0 (0%)	6938 (45%)
2	4128 (11%)	0 (0%)	4128 (30%)

© 2022 Bouchelle Z et al. *JAMA Health Forum.*

>=3	3288 (10%)	0 (0%)	3288 (26%)
SNAP receipt (yes or no)			
Yes	18106 (44%)	9882 (35%)	8224 (61%)
No	25934 (56%)	19804 (65%)	6130 (39%)
Free food receipt in previous 7 days (yes or no)			
Yes	6220 (16%)	3572 (13%)	2648 (20%)
No	37820 (84%)	26114 (87%)	11706 (80%)
Survey Wave Dates			
Jul 21-Aug 2, 2021	4077 (10%)	2793 (10%)	1284 (10%)
Aug 4-Aug 16, 2021	4174 (9%)	2812 (10%)	1362 (9%)
Aug 18-Aug 30, 2021	4143 (9%)	2829 (9%)	1314 (9%)
Sept 1-Sept 13, 2021	3874 (10%)	2665 (10%)	1209 (9%)
Sept 15-Sept 27, 2021	3648 (10%)	2494 (10%)	1154 (9%)
Sept 29-Oct 11, 2021	3525 (9%)	2441 (9%)	1084 (10%)
Dec 1-Dec 13, 2021	4445 (11%)	3038 (11%)	1407 (11%)
Dec 29, 2021-Jan 10, 2022	5431 (11%)	3507 (11%)	1924 (12%)
Jan 26-Feb 7, 2022	5248 (10%)	3464 (10%)	1784 (10%)
Mar 2-Mar 14, 2022	5475 (10%)	3643 (10%)	1832 (10%)
[a]Unweighted observation frequency.			
[b]Responses weighted using household survey weights divided by the 10 waves in sample.			
[c]The Household Pulse Survey does not collect additional information from respondents who identify as "any other race alone or in combination."			
Abbreviation: SNAP: Supplemental Nutrition Assistance Program			

© 2022 Bouchelle Z et al. *JAMA Health Forum.*

eMethods 1. Description of Difference-in-Differences Analysis

We used Stata 17.0 for our analyses and referenced the difference-in-differences technical documentation below.[1,2] We used the Stata's *didregress* command for our analyses. This command fits generalized difference-in-differences models, also known as two-way fixed effects models.[3] The difference-in-differences model is given by:

$$y_{ijt} = \gamma_j + \gamma_t + z_{ijt}\beta + D_{jt}\delta + \varepsilon_{ijt} \quad (1)$$

In our study, we have cross-sectional data for individuals representing households in different states, some of whom have children and some of whom do not. Thus, i denotes the individual/household, j the grouping of either being in a household with children or not, and t the survey wave. In (1) above, the model includes γ_j group fixed effects, γ_t time fixed effects, and a D_{jt} binary treatment variable (the interaction term) that varies at the group and time levels. In this case, the pre-treatment period includes survey waves during the period of distribution of monthly Child Tax Credit payments, July 15, 2021 to December 15, 2021. The post-treatment period includes survey waves that occurred after the discontinuation of monthly Child Tax Credit payments on December 15, 2021. z_{ijt} denotes the covariates and ε_{ijt} denotes the error term. β denotes the coefficients for each covariate. δ denotes the difference-in-difference estimate.[1,2]

We adjusted for covariates including gender at birth (female or male), age group (18-24, 25-44, or 45-65 years), race (Asian, Black or African American, White, or any other race alone or in combination), ethnicity (Hispanic, Latino, or Spanish origin or non-Hispanic, Latino, or Spanish origin), educational level (less than high school, high school or equivalent, some college or 2-year degree, or 4-year degree or graduate level degree), marital status (married or unmarried), number of adults in the household (1, 2, or ≥3), number of children in the household (0, 1, 2, or ≥3), Supplemental Nutrition Assistance Program (SNAP) receipt (yes or no), receipt of free food in previous 7 days (yes or no), annual income (<$25 000, $25 000-$34 999, or $35 000-$49 999), and state of residence. We clustered our standard errors at the state level.

© 2022 Bouchelle Z et al. *JAMA Health Forum.*

eMethods 2. Description of Parallel Trends Testing

For parallel trends testing, we utilized the Stata postestimation command *estat ptrends* to test whether the linear trends in the outcome variable were parallel between exposed and unexposed groups during the pretreatment period.[1,2] We referenced the difference-in-differences introduction and related documents to carry out our analyses and provide the description below.[1,2]

First, equation (1) above is simplified to:

$y_{ijt} = DID_{ijt} + \varepsilon_{ijt}$ (2)

Next, the linear-trends model was augmented with additional terms – $d_{t,0} = 1(dt = 0)$ is a variable indicating pretreatment time periods, $d_{t,1} = 1(d_t = 1)$ is a variable indicating posttreatment time periods, and w_i is a variable that is 1 if the individual belongs to an exposed group and is 0 if the individual belongs to an unexposed group. These terms and their interactions are included in equation 3:

$y_{ijt} = DID_{ijt} + w_i d_{t,0} t \zeta_1 + w_i d_{t,1} t \zeta_2 + \varepsilon_{ijt}$ (3)

In this model, the coefficient ζ_1 captures the differences in slopes between exposed and unexposed groups in pretreatment periods while ζ_2 captures the differences in slopes in posttreatment periods.[1]

Therefore, a ζ_1 of 0 suggests linear trends in the outcome are parallel during pretreatment periods. The Stata postestimation command *estat ptrends* uses a Wald test of ζ_1 against 0. The null hypothesis of this test is that the linear trends are parallel.[1] The results of this testing by income group are displayed below in eTable 6.

© 2022 Bouchelle Z et al. *JAMA Health Forum.*

eTable 6. Parallel Trends Testing for Difference-in-Differences Analyses, Stratified by Annual Income

Annual Income	Model	Prob > F
<$25 000	Unadjusted[a]	0.7424
	Adjusted[b]	0.4651
<$35 000	Unadjusted[a]	0.2428
	Adjusted[b]	0.6344
<$50 000	Unadjusted[a]	0.5432
	Adjusted[b]	0.9933

[a]Unadjusted model does not include covariate adjustment. Excludes respondents with missing data for primary dependent and independent variables in adjusted difference-in-differences analysis. Standard errors clustered at the state level.

[b]Adjusted model includes covariate adjustment for birth, age group, race, ethnicity, educational level, marital status, number of adults in the household, number of children in the household, Supplemental Nutrition Assistance Program (SNAP) receipt, receipt of free food in previous 7 days, annual income, and state of residence. Standard errors clustered at the state level.

© 2022 Bouchelle Z et al. *JAMA Health Forum.*

eMethods 3. Description of Event Study Specification

To assess for time specific-treatment effects, we also used an event study specification.[1] An event study allows us to estimate the association between the discontinuation of monthly CTC payments and household food insufficiency using a difference-in-differences approach that allows for the associations between exposure and outcome to vary over time.[3] We referenced the difference-in-differences introduction and related documents to carry out our analyses and provide the description below.[1,2] For the event study specification, we used the same difference-in-differences model described in eMethods 1:

$$y_{ijt} = \gamma_j + \gamma_t + z_{ijt}\beta + D_{jt}\delta + \varepsilon_{ijt} \ (1)$$

Next, we used the Stata postestimation command *estat grangerplot* to fit a model that includes lags and leads of an indicator for the time period in which the treatment began.[1] In this case, the pre-treatment period includes survey waves during the period of distribution of monthly Child Tax Credit payments, July 15, 2021 to December 15, 2021. The post-treatment period includes survey waves that occurred after the discontinuation of monthly Child Tax Credit payments on December 15, 2021.

The coefficients on the lags are used to assess whether there is any change in the treatment effect across posttreatment time periods.[1] The command *estat grangerplot* omits the first lead and sets the coefficient to 0 for the survey wave just prior to policy change, such that the plotted effects are normalized with respect to that time period.[1] The adjusted difference-in-differences estimates of the association between food insufficiency and discontinuation of monthly Child Tax Credit payments using an event study specification is shown below in eTable 7.

© 2022 Bouchelle Z et al. *JAMA Health Forum.*

eTable 7. Adjusted Difference-in-Differences Estimates of Association Between Food Insufficiency and Discontinuation of Monthly Child Tax Credit Payments, Event Study Specification

Annual income	Survey Wave	Leads and Lags	Coefficient	P-value	Lower bound 95% CI	Upper bound 95% CI
<$25 000	07/21/21	Lead 7	0.02	0.616	-0.05	0.08
	08/04/21	Lead 6	0.01	0.828	-0.07	0.09
	08/18/21	Lead 5	0.06	0.154	-0.02	0.14
	09/01/21	Lead 4	0.04	0.151	-0.02	0.10
	09/15/21	Lead 3	0.05	0.356	-0.05	0.15
	09/29/21	Lead 2	0.01	0.779	-0.05	0.07
	12/01/21	reference	0.00	ref	ref	ref
	12/29/21	Lag 0	0.09	0.033	0.01	0.16
	01/26/22	Lag 1	0.07	0.021	0.01	0.14
	03/02/22	Lag 2	0.10	0.000	0.05	0.16
<$35 000	07/21/21	Lead 7	-0.02	0.319	-0.07	0.02
	08/04/21	Lead 6	-0.02	0.589	-0.08	0.05
	08/18/21	Lead 5	0.03	0.306	-0.02	0.07
	09/01/21	Lead 4	0.00	0.966	-0.05	0.05
	09/15/21	Lead 3	0.01	0.750	-0.06	0.08
	09/29/21	Lead 2	-0.02	0.446	-0.07	0.03
	12/01/21	reference	0.00	ref	ref	ref
	12/29/21	Lag 0	0.03	0.278	-0.03	0.09
	01/26/22	Lag 1	0.06	0.022	0.01	0.12
	03/02/22	Lag 2	0.04	0.032	0.00	0.08
<$50 000	07/21/21	Lead 7	-0.01	0.564	-0.05	0.03
	08/04/21	Lead 6	-0.01	0.802	-0.05	0.04
	08/18/21	Lead 5	0.02	0.188	-0.01	0.06
	09/01/21	Lead 4	0.00	0.928	-0.04	0.05
	09/15/21	Lead 3	0.01	0.678	-0.04	0.07
	09/29/21	Lead 2	-0.02	0.452	-0.06	0.03
	12/01/21	reference	0.00	ref	ref	ref
	12/29/21	Lag 0	0.02	0.306	-0.02	0.07
	01/26/22	Lag 1	0.05	0.034	0.00	0.10
	03/02/22	Lag 2	0.03	0.051	0.00	0.07

Adjusted model includes covariate adjustment for birth, age group, race, ethnicity, educational level, marital status, number of adults in the household, number of children in the household, Supplemental Nutrition Assistance Program (SNAP) receipt, receipt of free food in previous 7 days. annual income, and state of residence. Standard errors are clustered at the state level. Responses weighted using household survey weights divided by the 10 waves in sample.

© 2022 Bouchelle Z et al. *JAMA Health Forum.*

eReferences

1. STATA. Postestimation tools for didregress and xtdidregress. Accessed April 3, 2022. https://www.stata.com/manuals/tedidregresspostestimation.pdf#tedidregresspostestimation
2. STATA. Introduction to difference-in-differences estimation. Accessed April 3, 2022. https://www.stata.com/manuals/tedidintro.pdf#teDIDintro
3. Goodman-Bacon A. Difference-in-differences with variation in treatment timing. *J Econom*. 2021;225(2):254-277. doi:10.1016/j.jeconom.2021.03.014

© 2022 Bouchelle Z et al. *JAMA Health Forum.*
